# Shared component modelling of early childhood anaemia and malaria in Kenya, Malawi, Tanzania and Uganda

**DOI:** 10.1186/s12887-022-03694-4

**Published:** 2022-11-03

**Authors:** Danielle J. Roberts, Temesgen Zewotir

**Affiliations:** School of Mathematics, Statistics and Computer Science, University of KwaZulu-Natal, Durban, South Africa

**Keywords:** Adjusted posterior odds ratios, Bayesian inference, Conditional autoregressive, Joint modelling, Spatial modelling

## Abstract

**Background:**

Malaria and anaemia contribute substantially to child morbidity and mortality. In this study, we sought to jointly model the residual spatial variation in the likelihood of these two correlated diseases, while controlling for individual-level, household-level and environmental characteristics.

**Methods:**

A child-level shared component model was utilised to partition shared and disease-specific district-level spatial effects.

**Results:**

The results indicated that the spatial variation in the likelihood of malaria was more prominent compared to that of anaemia, for both the shared and specific spatial components. In addition, approximately 30% of the districts were associated with an increased likelihood of anaemia but a decreased likelihood of malaria. This suggests that there are other drivers of anaemia in children in these districts, which warrants further investigation.

**Conclusions:**

The maps of the shared and disease-specific spatial patterns provide a tool to allow for more targeted action in malaria and anaemia control and prevention, as well as for the targeted allocation of limited district health system resources.

**Supplementary Information:**

The online version contains supplementary material available at 10.1186/s12887-022-03694-4.

## Background

Anaemia and malaria are major health concerns that cause considerable morbidity and mortality, especially among young sub-Saharan African children [1, 2]. Anaemia in children is a manifestation of many conditions, such as iron deficiency, malaria and chronic diseases, most of which are preventable and curable. This complex nature of anaemia makes it difficult to combat, as the cause needs to be treated, not just the symptom. However, in resource-limited settings, it is not always possible to identify the main cause of anaemia in the child as there are constraints to diagnosis, as well as treatment and prevention [3]. This highlights the significance of studies that investigate the relationship between anaemia and its various causes.

In malaria endemic regions, malaria is the main driver of childhood anaemia [4]. On the other hand, severe anaemia can increase a child’s susceptibility to malaria in these regions [5]. Malaria and anaemia share common risk factors, however, investigating the joint effect of such factors on each outcome has not been widely considered [5–7]. Both from an epidemiological and statistical view point, the advantages of applying models that combine information from related diseases has been well-documented [8]. Joint modelling also has several advantages over univariate analyses, which include improved control over Type I error rates during multiple testing and efficiency in estimating parameters [9]. Further, through joint modelling, the correlation between the outcomes can be quantified and controlled for. Several approaches to joint modelling exist. The most common approach is the use of a multivariate model, where the univariate models for each response are combined through the specification of a joint distribution for the random effects [9, 10]. Copula regression is another approach to simultaneously modelling multiple outcomes, where a copula function is used to separate the marginal distributions from the dependence structure of a given multivariate distribution [11].

Joint modelling can also be extended into disease mapping through spatial modelling. This aids in gaining more insight into the spatial variation of each of the multiple diseases, while accounting for the association between them. The contribution that a geographical location has on the risk of a disease serves as a surrogate for unmeasured risk factors. Spatial variation in these risk factors influences the patterns of disease risk and transmission [12]. Spatial mapping of single diseases is a well established method for identifying the geographical locations that are most at risk, thus creating a more effective delivery system of limited resources [12–16]. Such an approach for joint spatial modelling includes the multivariate conditional autoregressive (MCAR) model [17, 18]. This approach allows one to assess and visualise the residual spatial effect of the geographical location on each response, while controlling for the correlation between the responses. However, this MCAR approach does not allow one to assess how the correlation between the responses changes based on the geographical location.

The spatial extension of the copula approach to joint modelling of multiple responses can aid in answering questions about how the association between the responses varies according to the geographical location. However, a short-coming of the copula geoadditive model is that it is not able to inform us which geographical locations contribute to a higher or lower likelihood of both diseases simultaneously. This leads to a shared component model (SCM), in which the spatial effect is decomposed into a shared and disease-specific spatial effect. Therefore, this study considers a shared component model for the joint spatial analysis of anaemia and malaria in children in Kenya, Malawi, Tanzania and Uganda, where both the shared and disease-specific district-level spatial effects are estimated while controlling for known risk factors. This will allow the districts of high risk of one or the other, or both diseases to be identified for a more targeted approach to anaemia and malaria control and prevention as well as for a targeted allocation of limited district health system resources.

## Methods

### Data Source

Data from the Demographic and Health Surveys (DHS) and/or the Malaria Indicator Surveys (MIS) carried out in each of the four countries was used in this study. These surveys included the 2015 Kenya Malaria Indicator Survey, the 2017 Malawi Malaria Indicator Survey, the 2015-2016 Tanzania Demographic and Health Survey and Malaria Indicator Survey and the 2016 Uganda Demographic and Health Survey. While DHS data from different years is available for these countries, the data used here for each country was selected based on what was most recent at the time of conceptualising this study. The surveys were nationally represented and aimed at collecting data to monitor and evaluate population, health, and nutrition programs through numerous questionnaires. These surveys utilised a stratified two-stage cluster design where the first stage involved selecting clusters from a list of enumeration areas which made up the primary sampling units. Clusters were selected with a probability proportional to their size. The second stage involved systematic sampling of households from the list of households in each cluster, with an equal number of households selected from the clusters. The selected households were visited and interviewed by trained staff. In addition, biomarkers were collected from participants in selected households. A thorough review of the sampling methodology is presented in the DHS Sampling Manual [19]. In this study, we use the anaemia and malaria results of the blood specimens collected from a finger- or heel-prick of all children aged 6 to 59 months in the sampled households, with the consent of a parent or guardian.

### Study Variables

The dichotomised anaemia status and malaria status of the child were the two outcomes of interest. The child’s haemoglobin (Hb) concentration was measured using a portable HemoCue analyser, from which they were considered anaemic if their Hb level was under 11 g/dl after adjusting for altitude [20]. Using the SD Bioline Pf/Pv rapid diagnostic test (RDT), the presence of the *Plasmodium falciparum* parasite in the child’s blood was tested for. This *Plasmodium* species is the predominant cause of severe and fatal malaria in humans [21]. The child’s malaria status was based on this RDT result.

**Fig. 1:**
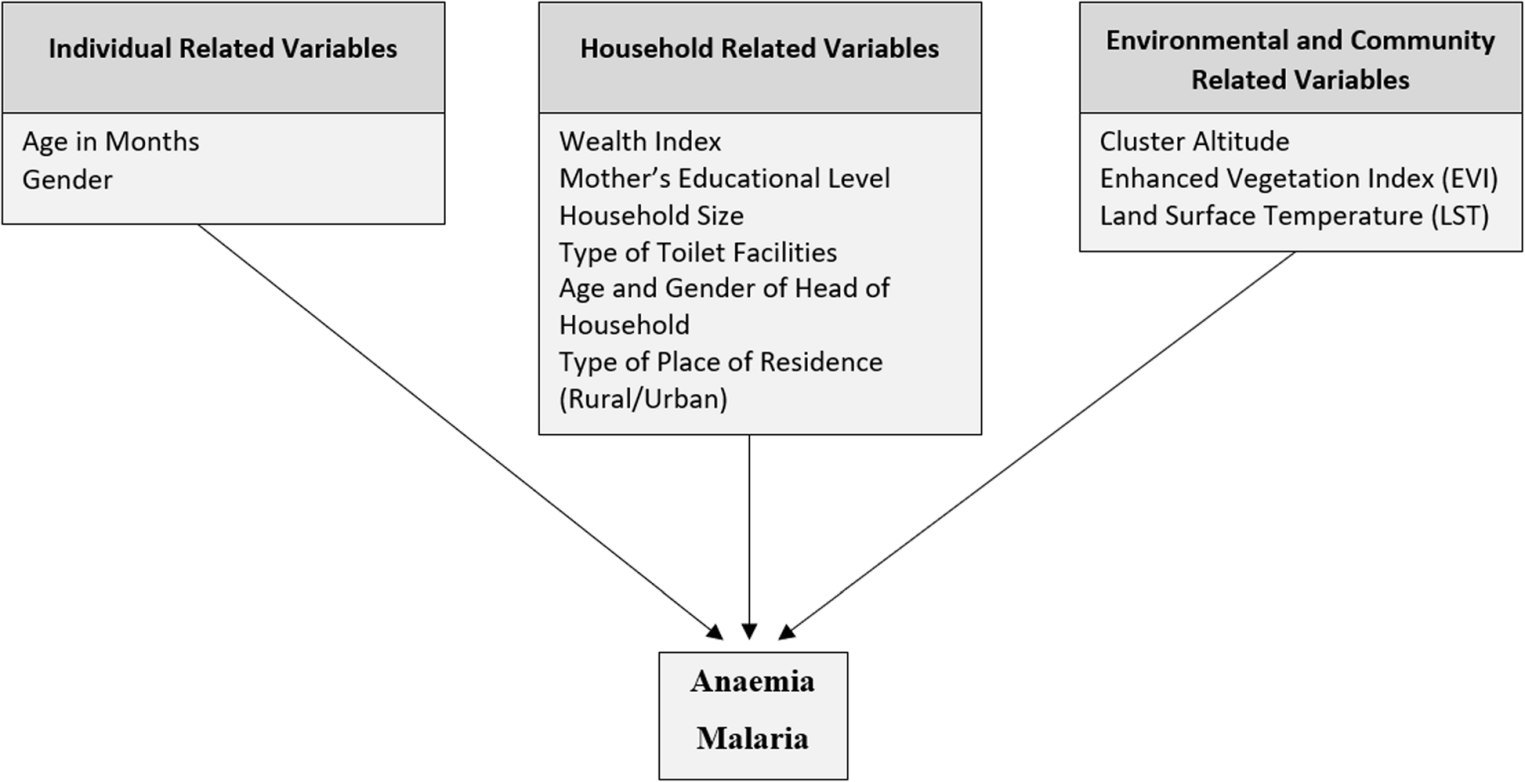
Potential risk factors of anaemia and malaria among young children

The covariates considered in this study included a range of demographic, socio-economic and environmental factors as presented in Fig. 1. These factors included the gender and age of the child, the mother’s highest education level, the number of members in the household (size of the household), the type of place of residence (rural or urban), the household wealth index Z-score, the type of toilet facility, the age and gender of the head of the household, and three environmental factors, namely the cluster altitude, day land surface temperature (LST) and the enhanced vegetation index (EVI). Anaemia and malaria transmission are both affected by various environmental factors, either directly or indirectly. EVI and LST serve as proxies for water-born intestinal parasites, which are contributors of childhood anaemia [22]. In addition, these environmental factors affect the *Plasmodium* parasite which causes malaria as well as the *Anopheles* mosquito which is the host for the parasite [23].

### Statistical Method

The shared component model (SCM) was originally proposed by Knorr-Held and Best [24] to jointly model the spatial variation of rates of several diseases with common risk factors. The SCM allows for the underlying risk surface of the diseases to be decomposed into two: shared and disease-specific variation. The SCM has been used in a wide variety of applications, such as to identify shared patterns among chronic related preventable hospitalizations [25], for joint spatial modelling of common morbidities of childhood fever and diarrhoea in Malawi [12], and for joint modelling of brain cancer incidence and mortality rates in two regions in the north of Spain [26]. Recently, the SCM was used to identify crime-general and crime-specific hotspots in a region in Canada [27].

The SCM is typically used when interest is on the relative risk of two or more diseases in a particular region, where regional level covariates can be incorporated in the model. In this case, the response represents the disease counts for the region. In this study, however, we consider the SCM to model the probability, $\pi _{ijk}$, of child *j* residing in district *i* having anaemia ($k=1$) or malaria ($k=2$). Thus, we make use of logistic regression models given by

1 $$\begin{aligned} logit(\pi _{ij1})&= \alpha _1 + \boldsymbol{x}^{\prime}_{ij1}\boldsymbol{\beta }_1+\delta u_i + v_{i1}, \end{aligned}$$

2 $$\begin{aligned} logit(\pi _{ij2})&= \alpha _2 + \boldsymbol{x}^{\prime}_{ij2}\boldsymbol{\beta }_2+\frac{u_i}{\delta } + v_{i2}, \end{aligned}$$

where $\alpha _k$, $k=1,2$, are the disease specific intercepts; $\boldsymbol{\beta }_k$ is the vector of regression parameters corresponding to the covariates $\boldsymbol{x}^{\prime}_{ijk}$ for the $k^{th}$ disease, where such covariates comprise of child-level, household-level and environmental factors; $u_i$ is the disease-general shared spatial component common to both diseases; and $v_{ik}$ is the disease-specific spatial component which captures the spatial patterns that deviate from the shared spatial component. Both the shared and specific spatial components were based on a total of 369 districts across the four countries. The parameter $\delta$ is referred to as the partitioning weight and allows for a different odds gradient of the shared component. Note that the weighted shared component and the disease-specific component add up to 100% of the spatial variation for each disease. The advantage of our approach to the SCM is that it enables one to explore the individual-, household-, and community-level risk factors for each disease. Thus, such risk factors are well accounted for in the model.

**Fig. 2:**
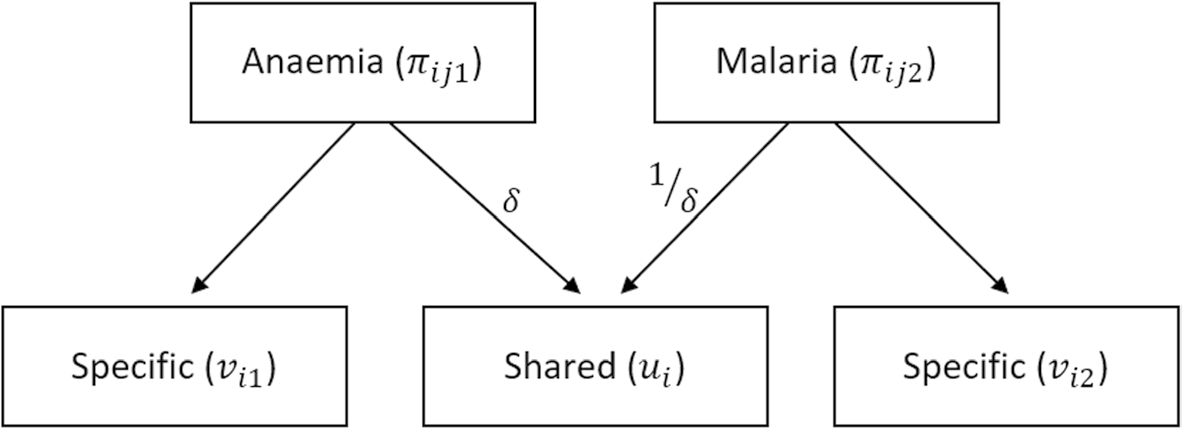
Schematic representation of the shared component model for this study

Figure 2 presents a schematic representation of the shared component model for this study. The shared component captures the spatial pattern common to both diseases, where $\delta$ allows each disease to have a unique association with this spatial pattern. A value of $\delta$ close to one indicates that anaemia and malaria have a similar magnitude of association with the shared spatial pattern, whereas a smaller positive value of $\delta$ indicates that anaemia has a weaker association with the shared spatial pattern compared to malaria [27]. It should be noted that estimating a partitioning weight ($\delta$) for one disease and assigning the inverse to the second disease improves model identifiability compared to estimating separate partitioning weights for each disease [24, 27]. In addition, only one parameter needs to be estimated, rather than two.

Each of the shared and disease-specific spatial components, $u_i$ and $v_{ij}$, can be decomposed as follows

$$\begin{aligned} u_i&=u_{str_i} + u_{unstr_i}, \\ v_{ij}&=v_{str_{ij}} + v_{unstr_{ij}}, \end{aligned}$$

where $u_{str_i}$ and $v_{str_{ij}}$ are spatially structured effects and $u_{unstr_i}$ and $v_{unstr_{ij}}$ are the random heterogeneity (spatially unstructured) effects. These spatial effects are due to unmeasured factors that have not been controlled for in the model, where such factors may be common among neighbouring districts (and thus contribute to the structured spatial effect) or specific to a district (and thus contribute to the unstructured spatial effect).

A Bayesian approach was used to fit the model, where each of the parameters were assigned a prior distribution. Weakly informative *N*(0, 10000) priors for the regression coefficients $\varvec{\beta }_k$ were assumed. The spatial components followed a Besag framework [13], where the structured spatial effects were assigned intrinsic Gaussian Markov random field (IGMRF) priors, also known as conditional autoregressive (CAR) priors. This prior assumes that the structured spatial effect of the districts follow a normal distribution with a conditional mean equal to the average of the neighbouring districts’ effects and a conditional variance inversely proportional to the number of neighbours. Two districts are considered neighbours if they share a border. The unstructured spatial effects were assigned i.i.d. Gaussian priors with a mean of zero. The variance components of these spatial effects comprised of unknown precision (inverse variance) parameters that were assigned a Gamma (1, 0.001) hyperprior distribution. The intercepts $\alpha _k$ were assigned flat priors as recommended for a model that includes a CAR random effect [28]. In addition, a sum-to-zero constraint was imposed on the spatial effects to allow for model identifiability. The partitioning weight $\delta$ was assigned a log-normal distribution with a mean of 0 and variance of 0.169 [24]. This prior then assumes that both $\delta$ and $1/\delta$ are both positive, which is a reasonable assumption as there is a positive correlation between anaemia and malaria (see [7]). This prior also assumes that the ratio of $\delta$ and $1/\delta$ (i.e. $\delta /(1/\delta )$) is between 0.2 and 5 with a 95% probability, regardless of which disease is labelled 1 or 2 [24].

The models were fitted using Markov Chain Monte Carlo (MCMC) simulations in WinBUGS version 1.4.3 [29]. The WinBUGS program for the area-level SCM was adapted for our child-level SCM. A copy of this adapted WinBUGS code is provided in Additional file 1. Three parallel MCMC chains with varying starting values were run for a total of 50 000 iterations each. After a burn-in period of 50 000, every 10th sample was retained for posterior inference. Convergence was assessed using the Brooks and Gelman statistic and autocorrelation plots. A sensitivity analysis with various prior and hyperprior specifications was performed. The estimates and their significance remained largely the same. The models were compared using the deviance information criterion (DIC), where the results presented in this study are based on the model with the lowest DIC. The estimated spatial effects were extracted and mapped in QGIS 3.20 (https://qgis.org/en/site/index.html). All of the maps created were based on the results of this study and made use of shapefiles freely available from the DHS Program’s Spatial Data Repository (https://spatialdata.dhsprogram.com/boundaries).

### Ethics

Ethics approval for the primary study in each of the four countries were granted by the relevant Ethics Review Committee and ICF International’s Institutional Review Board within each country. All methods were carried out in accordance with relevant guidelines and regulations.

## Results

### Sample Characteristics

**Fig. 3:**
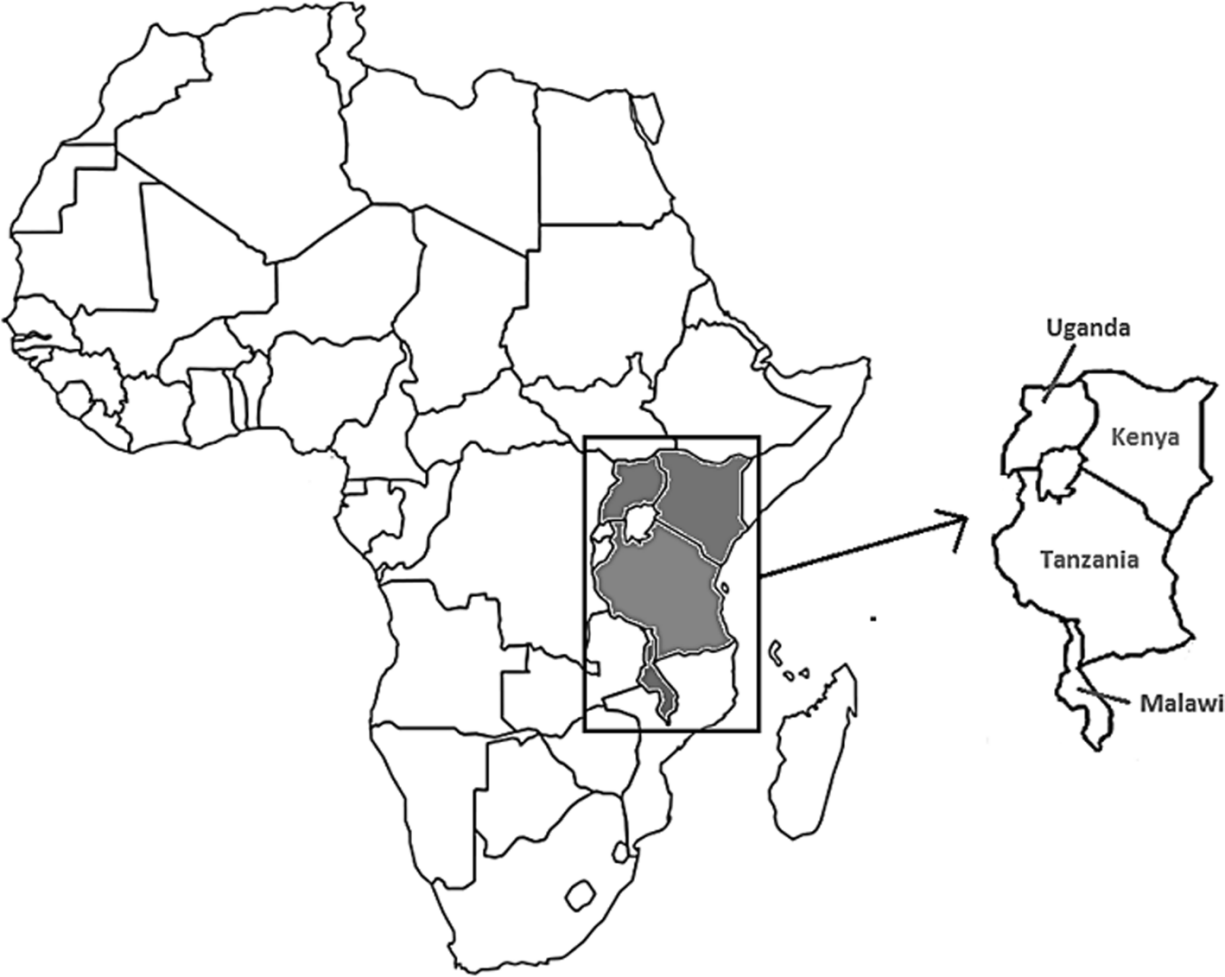
Study area

The final data set in this study comprised of 18196 children from across the four countries, with 12.5% from Malawi, 18.8% from Kenya, 25.7% from Uganda and 43% from Tanzania. These countries form one contiguous region (Fig. 3), which was partitioned according to the districts of each country. The 369 districts considered in this study comprised of all 47 counties or districts from Kenya; 26 out of 28 districts for which data was available from Malawi; 175 out of 184 districts for which data was available from mainland Tanzania; and 121 out of 122 districts for which data was available for Uganda.

The observed prevalence of malaria was 19.7%, while anaemia was more prevalent at 52.5%. A total of 15.1% of the sampled children had both anaemia and malaria. More detailed sample descriptives are presented in our previous studies [7, 14, 30].

### Fixed effects results of the shared component model

Table 1 presents the adjusted posterior odds ratios (AOR) and corresponding 95% credible intervals for the fixed effects. It should be noted that our previous study revealed a non-linear effect of the child’s age in months on the likelihood of anaemia [7], where there was an increase in the likelihood for children younger than 12 months followed by a decrease in the likelihood for children aged 12 months and older. However, due to the limitations of WinBUGS, in this study the effect of age was incorporated as a linear fixed effect where it was categorised accordingly (under 12 months versus 12 months and older). The child’s age had a significant effect on the likelihood of anaemia as well as malaria. However, while the odds of anaemia were substantially lower for those aged 12 months and older (AOR = 0.316; 95% CrI: 0.285-0.351), the odds of malaria were higher for children in this age group compared to those younger than 12 months (AOR = 2.166; 95% CrI: 1.850-2.531). The odds of anaemia were significantly lower for female children compared to male children (AOR = 0.879; 95% CrI: 0.826-0.936). However, there was no significant difference in the odds of malaria between male and female children (AOR = 0.977; 95% CrI: 0.892-1.068). The type of place of residence only had a significant effect on the likelihood of malaria in children, not anaemia, where those residing in rural areas were 1.797 times more likely to have malaria compared to those residing in urban areas (95% CrI: 1.514-2.136). The mother’s highest educational level had a significant impact on the odds of anaemia as well as malaria, where the odds of each decreased with an increase in education level. Likewise, there was a significant decrease in the odds of either disease with an increase in the household’s wealth index Z-score (AOR = 0.769; 95% CrI: 0.725-0.816 for anaemia, AOR = 0.400; 95% CrI: 0.360-0.443 for malaria). In addition, there was a significant decrease in the odds of anaemia with an improvement in toilet facilities. However, the type of toilet facility had no significant effect on the odds of malaria in children. The cluster altitude had a decreased effect on both the odds of anaemia and malaria (AOR = 0.969; 95% CrI: 0.953-0.985 for anaemia, AOR = 0.847; 95% CrI: 0.819-0.874 for malaria). The odds of malaria significantly increased with an increase in the environmental factor EVI (AOR = 2.074; 95% CrI: 1.380-3.304), however it had no significant effect on the odds of anaemia (AOR = 1.050; 95% CrI: 0.889-1.268). The gender of the head of household, household size and the environmental factor LST did not have any significant effects on the odds of anaemia or malaria.

**Table 1:** Adjusted posterior odds ratio estimates (AOR) and 95% credible intervals

Variable	Anaemia	Malaria
	AOR (95% CrI)	AOR (95% CrI)
*Gender (ref = Male)*		
Female	0.879 (0.826, 0.936)^a^	0.977 (0.892, 1.068)
*Age in Months (ref = Under 12 months)*		
12 months and older	0.316 (0.285, 0.351)^a^	2.166 (1.850, 2.531)^a^
*Type of Place of Residence (ref = Urban)*		
Rural	0.948 (0.859, 1.047)	1.797 (1.514, 2.136)^a^
*Mother’s Education Level (ref = No Education)*		
Primary	0.874 (0.793, 0.964)^a^	0.803 (0.703, 0.915)^a^
Secondary and Higher	0.852 (0.748, 0.972)^a^	0.609 (0.498, 0.749)^a^
Unknown	0.742 (0.654, 0.838)^a^	1.119 (0.946, 1.329)
*Gender of Household Head (ref = Male)*		
Female	1.005 (0.931, 1.082)	0.927 (0.828, 1.038)
*Type of Toilet Facility (ref = No Facilities)*		
PIT Latrine	0.779 (0.697, 0.869)^a^	0.878 (0.757, 1.017)
Flush Toilet	0.763 (0.624, 0.929)^a^	1.051 (0.667, 1.620)
*Household Size*	1.008 (0.998, 1.019)	1.003 (0.990, 1.016)
*Wealth Index*	0.769 (0.725, 0.816)^a^	0.400 (0.360, 0.443)^a^
*Cluster Altitude (in 100 metres)*	0.969 (0.953, 0.985)^a^	0.847 (0.819, 0.874)^a^
*EVI*	1.050 (0.889, 1.268)	2.074 (1.380, 3.304)^a^
*LST*	1.011 (0.969, 1.055)	1.037 (0.922, 1.180)

^a^significant at 5% level of significance

The estimates for the shared and disease-specific spatial components for each district are provided in Additional file 2. However, for convenience, these estimates have been illustrated in maps which are discussed below.

### Spatial effects results of the shared component model

**Fig. 4:**
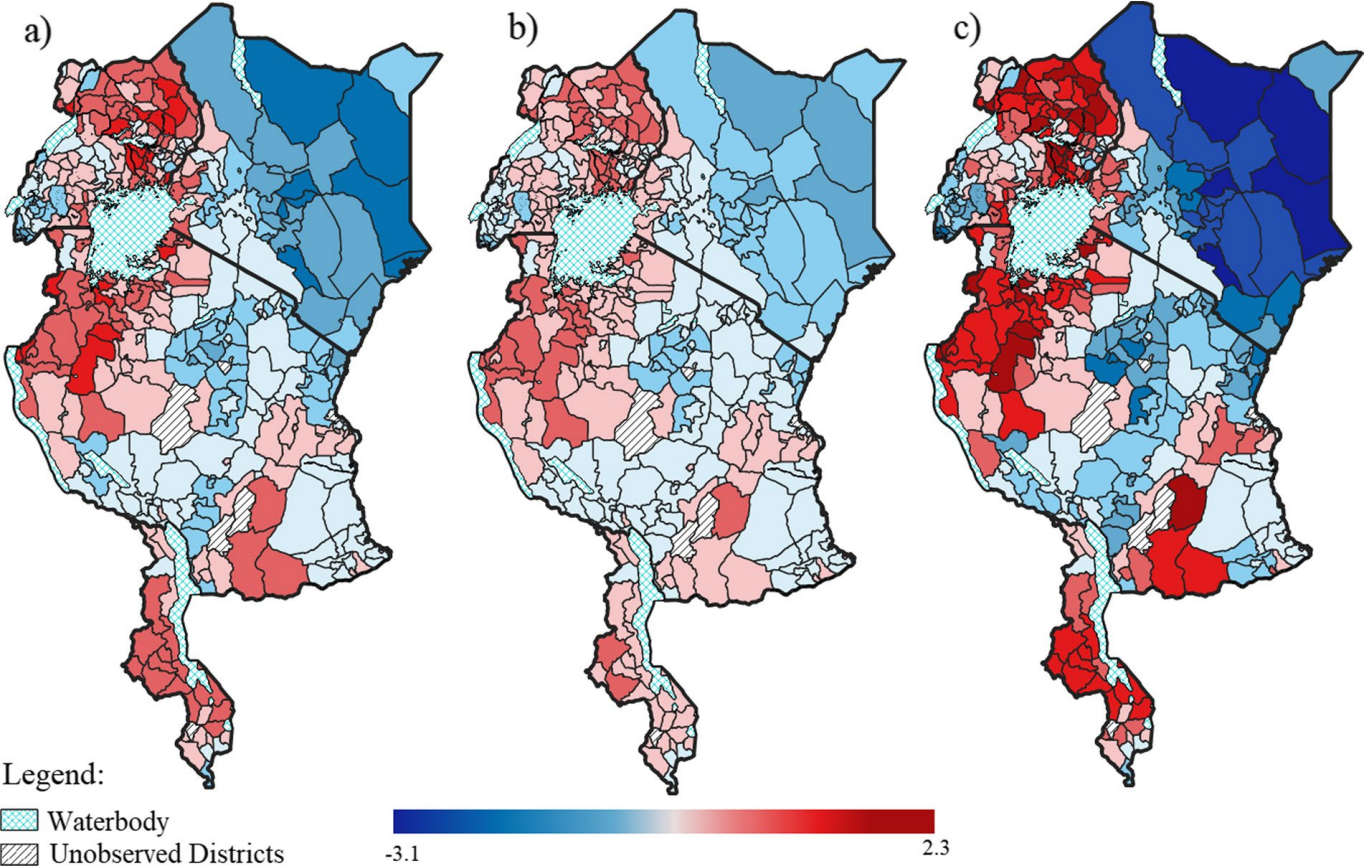
Estimated effect of the shared spatial component (**a**); shared spatial component for anaemia (**b**); and shared spatial component for malaria (**c**)

Figure 4a presents the estimated effect of the shared spatial component on the log-odds of anaemia and malaria. The districts in blue shadings correspond to a negative estimated log-odds and were therefore associated with a lower likelihood of the disease. Whereas, those in red shadings correspond to a positive estimated log-odds and were therefore associated with a higher likelihood. Notably, there were distinct patterns of clustering among neighbouring districts. In particular, there were clusters associated with increased likelihoods of both diseases in the west of Tanzania and throughout Uganda and Malawi. Kenya primarily consisted of districts/counties associated with decreased likelihoods of both diseases. This shared spatial effect presented a non-random pattern, as suggested by Moran’s *I* statistic of 0.758 (*p* = 0.001). The partitioning weight ($\delta$) was estimated at 0.626 (95% CrI: 0.429-0.952) (Table 2). Thus, malaria had a stronger association with this shared spatial pattern compared to anaemia, as is evident from the shared spatial effects in Fig. 4b and 4c for anaemia and malaria, respectively. Table 2 indicates that 82.70% of the spatial variation in the likelihood of anaemia was captured by the shared spatial component, while only 62.44% of the spatial variation in the likelihood of malaria was captured by this component.

**Table 2:** Partitioning weight posterior estimate (95% CrI) and empirical variances

	Anaemia	Malaria
Partitioning weight ($\delta$)	0.626 (0.429, 0.952)	1.597 (1.050, 2.331)
Empirical variance of shared component	0.182	1.183
Empirical variance of disease-specific component	0.083	0.712
% of total variation explained by shared component	82.70	62.44

**Fig. 5:**
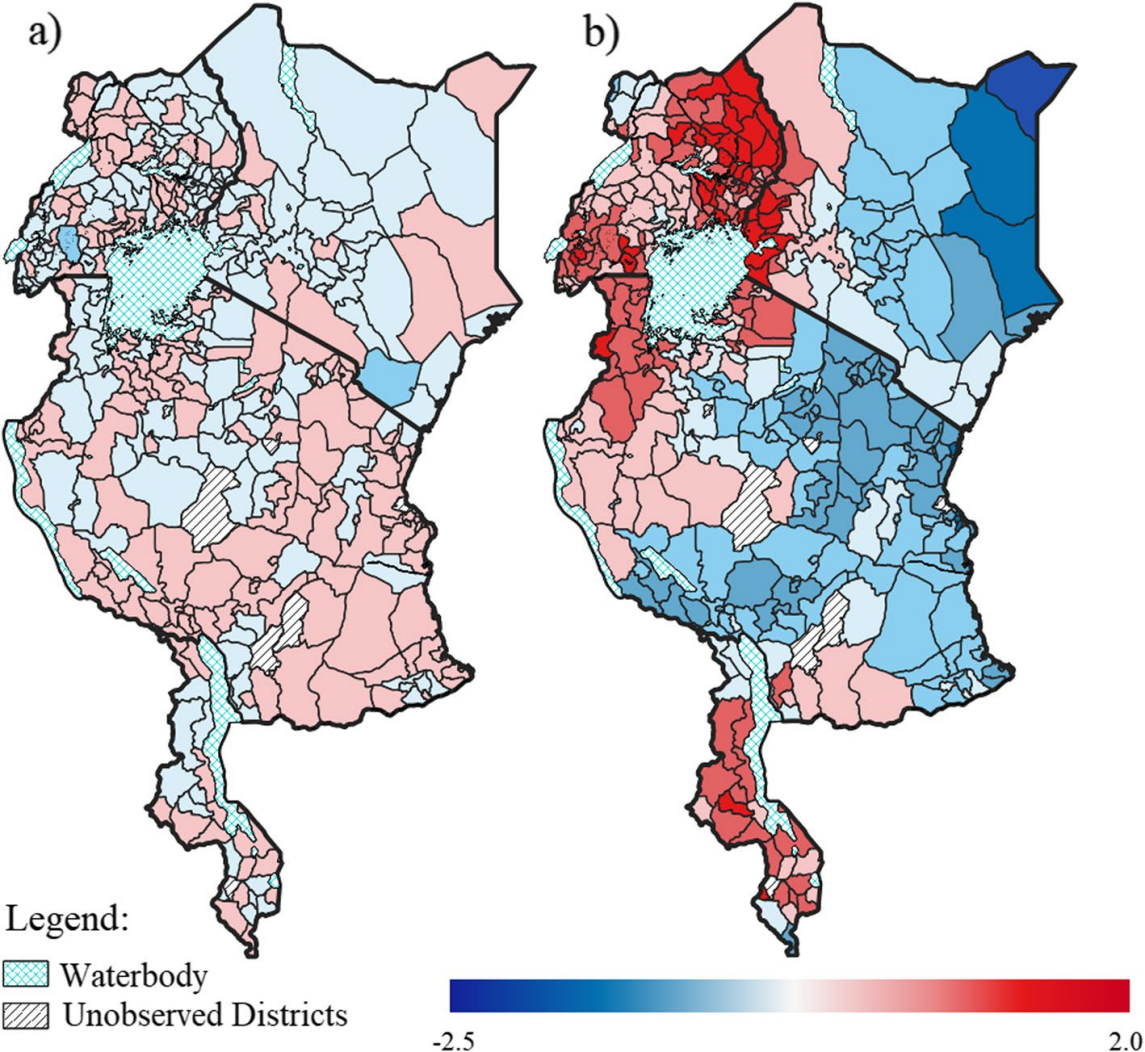
Estimated effect of the disease-specific component for anaemia (**a**) and the disease-specific component for malaria (**b**)

The disease-specific spatial effects for anaemia and malaria are displayed in Fig. 5a and 5b, respectively. Similar to the shared spatial component, this disease-specific spatial effect was more prominent for malaria than for anaemia. In addition, this component explained a higher proportion of the spatial variation in the likelihood of malaria (37.56%) compared to that for anaemia (17.30%). Once again, both spatial patterns consisted of clusters of increased likelihoods (positive values) and decreased likelihoods (negative values). These patterns were non-random, as confirmed by Moran’s *I* statistic of 0.258 for anaemia and 0.866 for malaria, both of which were significant at a 5% level of significance. Unlike the shared component, there were fewer clusters in the west of Tanzania and in Uganda for the anaemia-specific spatial effect. Multiple districts (229 out of 369 districts) across the four countries had contrasting effects on the likelihood of anaemia and malaria. More specifically, many of the districts that had a decreased likelihood of malaria, had an increased likelihood of anaemia.

## Discussion

This study aimed at jointly modelling the spatial variation in the likelihood of anaemia and malaria in young children across the districts of Kenya, Malawi, Tanzania and Uganda, while controlling for child-level, household-level and environmental characteristics. The spatial variation was considered at district level as the districts represent the administrative level for which public health decisions are implemented within the four countries. The district-level spatial effect for each disease was partitioned into a shared spatial component and a disease-specific spatial component. These spatial components can be considered as proxies for variations in unmeasured factors that contribute to both (shared) or only one (specific) of the diseases [27]. In this study, each of the shared and disease-specific spatial components were further partitioned into structured and unstructured spatial effects to account for unmeasured factors that are shared among neighbouring districts or that are district-specific, respectively.

The shared spatial component is due to the common effects of unmeasured shared risk factors. Malaria had a stronger association with the shared spatial component compared to anaemia, as evident by the higher partitioning weight. This suggests that the unmeasured risk factors common to both diseases had a higher impact on the likelihood of malaria. The shared spatial pattern revealed significant hotspots of increased likelihoods of each disease in the west of Tanzania and throughout the majority of the districts in Uganda and Malawi. This shared spatial component had a higher contribution to the spatial variation in the likelihood of both diseases compared to the disease-specific spatial component. This suggests that if programs for control and prevention of one of the diseases are targeted in the high risk districts, they should also make an impact on the other disease.

The disease-specific component was more prominent for malaria as well as contributed to a higher proportion of the spatial variation in the likelihood of malaria compared to that of anaemia. This indicates that there are additional unmeasured risk factors relevant to malaria only. One of the consequences of malaria is anaemia [31]. However, while severe anaemia can exacerbate malaria, it does not lead to malaria [5]. It is therefore reasonable to hypothesize that there are other drivers of anaemia in children in the districts that are associated with an increased likelihood of anaemia but a decreased likelihood of malaria based on the disease-specific spatial component. This study identified multiple of these districts throughout all four countries. Such drivers may include those that have a direct effect on anaemia but not malaria, such as iron deficiency, sickle cell anaemia and intestinal parasites, some of which have been shown to be protective against malaria [31–34].

Of note from the malaria-specific spatial pattern is that the districts with increased likelihoods were clustered around many of the water bodies in the countries, such as Lake Victoria shared by Tanzania, Kenya and Uganda, Lake Malawi, and Lake Turkana in Kenya. It has been suggested that the lake environments, specifically wetlands along the lakeshore, may maintain a high number of malaria vectors [35]. In particular, several vector breeding sites have been found to be associated with Lake Victoria and Lake Malawi [35, 36]. Thus, efforts for malaria vector control, such as insecticide-treated nets and indoor residual spraying, should be continued and up-scaled in these high risk districts. Such control measures have been noted as the primary driver of the significant reductions in the burden of malaria in sub-Saharan Africa over the past two decades [37]. This clustering pattern of increased likelihood of malaria around the water bodies differed for the anaemia-specific spatial component, which had less distinctive patterns. Anaemia is likely driven more by demographic, socioeconomic, and dietary-related factors than environmental factors, as suggested by the fixed effects results in this study, as well as highlighted in other studies which found malnutrition and intestinal parasites to also play a role in childhood anaemia [38–40].

While the focus of this study was not on determining the significant risk factors of each disease, the SCM allowed us to identify as well as control for such. However, the findings of this study regarding the child-level, household-level and environmental factors largely agreed with that of our previous study, which provides a detailed discussion on each [7]. In summary, the child’s age, the mother’s education level, the household wealth index, and cluster altitude had a significant effect on the likelihood of both anaemia and malaria. While the type of place of residence was not significantly associated with a child’s anaemia status, those residing in rural areas had a significantly higher likelihood of having malaria. This common finding has resulted in malaria being considered predominantly as a rural disease in Africa [41]. In rural areas, poor-quality household construction materials are common, which have been shown to be associated with a higher incidence of malaria due to increased mosquito entry [42, 43].

This study was limited with the amount of data available on the possible risk factors of either of the diseases, which may have had an influence on the spatial effects. In addition, the data is cross-sectional in nature, and thus no causal effect can be concluded. Another limitation to the study includes the dichotomisation of the child’s anaemia status which may result in a loss of information. However, using the ordinal form of anaemia (non-anaemic, mild, moderate and severe) would have restricted the statistical approach considered as the shared component model would not be appropriate. The strength of this study lies in the novelty of applying a child-level shared component model with district-level shared and disease-specific spatial effects to model the likelihood of anaemia and malaria in a child, which, to our knowledge, has not been considered for these two diseases. This individual-level covariate-adjusted approach has aided in identifying districts with an increased likelihood of either both or only one of the diseases, as presented by the shared and disease-specific spatial maps, respectively. These maps provide a tool to allow for more targeted action, either in the form of further investigation into the particular districts or in the form of programs and interventions, as well as the targeted allocation of limited district health system resources.

## Conclusion

As it is more common for co-infection to start with malaria, we recommend that programs and interventions for malaria in children be targeted in high malaria risk districts as identified by both the shared and malaria-specific spatial components in this study, which would likely also make a positive impact on anaemia. Moreover, further investigation into those districts with simultaneous high anaemia risk and low malaria risk should be considered in order to identify the significant drivers of anaemia in children within those districts. This would aid in applying the appropriate control measures and interventions for childhood anaemia in those districts, while saving on resources for malaria control and prevention which should be directed to the districts most in need.

## Supplementary Information

**Additional file 1.**

**Additional file 2.**

## Data Availability

This study utilised existing survey datasets that are in the public domain and freely available from http://www.dhsprogram.com/data/dataset_admin/login_main.cfm with the permission from the DHS Program.

## Abbreviations

95% CrI: 95% credible intervals
AOR: Adjusted odds ratio
CAR: Conditional autoregressive
DHS: Demographic and Health Survey
EVI: Enhanced vegetation index
Hb: Haemoglobin
IGMRF: Intrinsic Gaussian Markov random field
MCAR: Multivariate conditional autoregressive
MCMC: Markov Chain Monte Carlo
MIS: Malaria Indicator Survey
RDT: Rapid diagnostic test
SCM: Shared component model
WHO: World Health Organization
LST: Land surface temperature
