# Supplementary material for: Shared component modelling of early childhood anaemia and malaria in Kenya, Malawi, Tanzania and Uganda
Source: BMC Pediatr. 2022 Nov 3;22:631. doi: 10.1186/s12887-022-03694-4 (PMC9632052; doi:10.1186/s12887-022-03694-4)
Supplement: Supplementary file 1 — Additional file 1. [file 12887_2022_3694_MOESM1_ESM.pdf]

Additional File 1: Shared component modelling of early childhood anaemia and malaria in Kenya, Malawi, Tanzania and Uganda

WinBUGS Child-level SCM Code:

```
model
 {
   for (i in 1:2){
 for(j in 1:N){# number of children
            y[i,j] ~ dbern(p[i,j])

            logit(p[i,j]) <-  beta[i,1]  + beta[i,2]*Age_Above[i,j] +  beta[i,3]*GenderFemale[i,j]  +
beta[i,4]*EduLevel_Prim[i,j] + beta[i,5]*EduLevel_SecHigh[i,j] +  beta[i,6]*EduLevel_Unk[i,j] +
beta[i,7]*ToiletFacPIT[i,j] + beta[i,8]*ToiletFacFlush[i,j] + beta[i,9]*HHGender_Female[i,j] +
beta[i,10]*TypeResRural[i,j] + beta[i,11]*NumHHMembers[i,j] +
beta[i,12]*WealthIndexZScore[i,j] + beta[i,13]*ClusterAltitude [i,j] +  beta[i,14]*LST [i,j] +
beta[i,15]*EVI[i,j] + spat[District[i,j],i]
                        }}

# Spatial effect (decomposed into shared and specific)
             for (k in 1:369){
            spat[k,1] <- shared[k]*delta + specific[1,k]
            spat[k,2] <- shared[k]/delta + specific[2,k]
                        }

# Shared component:
            for (k in 1:369){
            shared[k] <- structshared[k] + unstructshared[k]
            unstructshared[k] ~dnorm(0,tauinshared)
                                    }

            structshared[1:369] ~car.normal(adj[],weights[],num[],taushared)



# Specific component:
             for (i in 1:2){
             for (k in 1:369){
               specific[i,k] <- struct[i,k] + unstruct[i,k]
                        unstruct[i,k] ~dnorm(0,tauin[i])
                                    }
                        struct[i,1:369] ~car.normal(adj[],weights[],num[],tau[i])
                                    }
```

```
# Hyperpriors for Precision Parameters:

        # Precision parameters for shared spatial component
                taushared ~dgamma(1,0.001)
                tauinshared ~dgamma(1,0.001)

        # Precision parameters for specific spatial component
                for (i in 1:2){
                tau[i] ~dgamma(1,0.001)
                tauin[i] ~dgamma(1,0.001)
                      }

# weights for spatial CAR effect
                for (k in 1:sumNumNeigh){
                        weights[k] <-1
                            }

# prior for delta:
         logdelta ~ dnorm(0,5.9)
         delta <- exp(logdelta)

 # vague prior on regression parameters
   for (i in 1:2) {
                for (k in 2:15){
                beta[i,k]~ dnorm(0, 1.0E-04)
                        }

 # Flat prior on intercept
          beta[i,1]~ dflat()
                        }

# Empirical Variances
        for (i in 1:369){
                anaemia.shared[i] <- shared[i]*delta
                malaria.shared[i] <- shared[i]/delta
                        }

        shared.var[1] <- sd(anaemia.shared[])*sd(anaemia.shared[]) # for anaemia
        shared.var[2] <- sd(malaria.shared[])*sd(malaria.shared[]) # for anaemia

        specific.var[1] <- sd(specific[1,])*sd(specific[1,])            # for anaemia
        specific.var[2] <- sd(specific[2,])*sd(specific[2,])            # for malaria
```

```
# Proportion of variation in log-likelihood of each disease explained by shared component:

	prop.shared[1] <-	shared.var[1]/(shared.var[1]+specific.var[1]) # for anaemia
	prop.shared[2] <-	shared.var[2]/(shared.var[2]+specific.var[2]) # for malaria

	}
```
