# Supplementary material for: Shared component modelling of early childhood anaemia and malaria in Kenya, Malawi, Tanzania and Uganda
Source: BMC Pediatr. 2022 Nov 3;22:631. doi: 10.1186/s12887-022-03694-4 (PMC9632052; doi:10.1186/s12887-022-03694-4)
Supplement: Supplementary file 2 — Additional file 2. [file 12887_2022_3694_MOESM2_ESM.pdf]

Additional file 2: Shared and disease-specific spatial component estimates by district

District	Country	Shared Component	Anaemia Shared Component	Malaria Shared Component	Anaemia Specific Component	Malaria Specific Component
Baringo	Kenya	-0.755	-0.473	-1.205	-0.029	-0.143
Bomet	Kenya	-0.804	-0.504	-1.283	-0.093	-0.090
Bungoma	Kenya	0.388	0.243	0.619	-0.141	1.171
BusiaK	Kenya	0.206	0.129	0.330	-0.441	1.293
Elgeyo-Marakwet	Kenya	-0.357	-0.223	-0.570	-0.105	0.290
Embu	Kenya	-1.382	-0.866	-2.206	-0.031	-0.808
Garissa	Kenya	-1.652	-1.035	-2.637	0.033	-1.510
Homa Bay	Kenya	0.225	0.141	0.359	-0.223	1.106
Isiolo	Kenya	-1.273	-0.797	-2.032	-0.022	-0.878
Kajiado	Kenya	-0.225	-0.141	-0.359	0.374	-0.456
Kakamega	Kenya	0.553	0.346	0.883	0.105	1.077
Kericho	Kenya	-0.689	-0.432	-1.100	-0.238	0.236
Kiambu	Kenya	-0.867	-0.543	-1.384	-0.207	-0.206
Kilifi	Kenya	-1.045	-0.655	-1.668	-0.325	-0.131
Kirinyaga	Kenya	-1.526	-0.956	-2.436	-0.162	-0.777
Kisii	Kenya	-0.253	-0.159	-0.404	-0.336	0.804
Kisumu	Kenya	0.332	0.208	0.530	-0.131	1.215
Kitui	Kenya	-1.361	-0.853	-2.173	-0.019	-0.860
Kwale	Kenya	-0.755	-0.473	-1.205	-0.292	-0.083
Laikipia	Kenya	-1.181	-0.740	-1.885	-0.100	-0.580
Lamu	Kenya	-1.512	-0.947	-2.414	-0.046	-1.259
Machakos	Kenya	-1.282	-0.803	-2.047	-0.120	-0.646
Makueni	Kenya	-1.566	-0.981	-2.500	-0.342	-0.642
Mandera	Kenya	-0.830	-0.520	-1.324	0.414	-2.352
Marsabit	Kenya	-1.908	-1.195	-3.046	-0.269	-0.984
MeruK	Kenya	-1.610	-1.009	-2.570	-0.257	-0.862
Migori	Kenya	0.580	0.363	0.926	-0.194	1.217
Mombasa	Kenya	-1.569	-0.983	-2.505	-0.204	-0.551
Murang'a	Kenya	-1.337	-0.837	-2.134	-0.151	-0.618
Nairobi	Kenya	-1.178	-0.738	-1.881	-0.204	-0.459
Nakuru	Kenya	-0.336	-0.210	-0.536	-0.082	0.127

Nandi	Kenya	-0.601	-0.376	-0.959	-0.278	0.477
Narok	Kenya	-0.054	-0.034	-0.087	0.101	-0.097
Nyamira	Kenya	-0.877	-0.549	-1.400	-0.311	0.249
Nyandarua	Kenya	-0.916	-0.574	-1.462	-0.008	-0.418
Nyeri	Kenya	-1.234	-0.773	-1.970	0.011	-0.723
Samburu	Kenya	-1.360	-0.852	-2.171	-0.073	-0.676
Siaya	Kenya	0.565	0.354	0.903	-0.288	1.651
Taita Taveta	Kenya	-1.058	-0.663	-1.689	-0.571	-0.393
Tana River	Kenya	-1.424	-0.892	-2.273	0.178	-1.244
Tharaka-Nithi	Kenya	-1.548	-0.970	-2.471	-0.118	-0.884
Trans Nzoia	Kenya	-0.170	-0.106	-0.271	-0.221	0.666
Turkana	Kenya	-1.282	-0.803	-2.047	-0.484	0.004
Uasin Gishu	Kenya	-0.228	-0.143	-0.364	0.204	0.106
Vihiga	Kenya	0.066	0.041	0.105	-0.289	1.225
Wajir	Kenya	-1.757	-1.101	-2.805	-0.083	-1.584
West Pokot	Kenya	0.031	0.020	0.050	0.108	0.524
Balaka	Malawi	0.310	0.194	0.495	0.006	0.316
Chikwawa	Malawi	0.123	0.077	0.197	0.357	-0.404
Chiradzulu	Malawi	0.139	0.087	0.222	-0.281	0.894
Chitipa	Malawi	0.272	0.170	0.434	0.156	-0.293
Dedza	Malawi	0.726	0.455	1.159	0.011	0.674
Dowa	Malawi	0.664	0.416	1.060	-0.236	1.045
Karonga	Malawi	0.099	0.062	0.157	0.003	-0.253
Kasungu	Malawi	0.855	0.536	1.365	-0.024	0.874
Machinga	Malawi	0.657	0.412	1.049	0.181	0.323
Mangochi	Malawi	0.675	0.423	1.078	0.033	0.561
Mchinji	Malawi	0.740	0.464	1.182	0.119	0.440
Mulanje	Malawi	-0.307	-0.192	-0.489	-0.347	0.604
Mwanza	Malawi	0.320	0.200	0.510	-0.242	1.008
Nkhata Bay	Malawi	0.396	0.248	0.633	-0.067	0.508
Nkhotakota	Malawi	0.667	0.417	1.064	0.088	0.542
Nsanje	Malawi	-0.562	-0.352	-0.898	0.208	-1.144
Ntcheu	Malawi	0.703	0.441	1.123	-0.064	0.788
Ntchisi	Malawi	0.695	0.435	1.110	-0.011	0.744
Phalombe	Malawi	0.150	0.094	0.239	0.017	0.453

Rumphi	Malawi	-0.131	-0.082	-0.209	-0.062	-0.133
Salima	Malawi	0.548	0.343	0.875	0.105	0.407
Thyolo	Malawi	-0.062	-0.039	-0.099	-0.152	0.273
Zomba	Malawi	0.541	0.339	0.864	-0.131	0.896
Blantyre	Malawi	0.480	0.301	0.767	0.047	0.545
Lilongwe	Malawi	0.809	0.507	1.291	0.021	0.731
Mzimba	Malawi	0.506	0.317	0.808	-0.044	0.606
Ukerewe	Tanzania	0.814	0.510	1.300	0.051	0.631
Songea DC	Tanzania	0.456	0.286	0.728	0.017	0.461
Arusha Urban	Tanzania	-0.018	-0.011	-0.029	0.281	-1.366
Karatu	Tanzania	-0.669	-0.419	-1.068	0.010	-0.835
Longido	Tanzania	-0.301	-0.188	-0.480	0.375	-1.176
MeruT	Tanzania	-0.584	-0.366	-0.932	-0.002	-1.314
Monduli	Tanzania	-0.712	-0.446	-1.137	-0.088	-1.046
Ngorongoro	Tanzania	-0.034	-0.021	-0.054	0.412	-0.502
Ilala	Tanzania	0.054	0.034	0.087	0.429	-1.579
Kinondoni	Tanzania	-0.378	-0.237	-0.604	0.201	-1.683
Kigamboni	Tanzania	-0.229	-0.143	-0.365	0.097	-1.225
Bahi	Tanzania	-0.949	-0.595	-1.515	-0.174	-0.852
Chamwino	Tanzania	-0.536	-0.336	-0.856	0.147	-1.066
Chemba	Tanzania	-0.829	-0.520	-1.324	-0.119	-0.866
Dodoma Urban	Tanzania	-0.433	-0.271	-0.692	0.271	-1.281
Kongwa	Tanzania	-0.433	-0.271	-0.690	0.128	-1.185
Mpwapwa	Tanzania	-0.445	-0.278	-0.710	0.158	-1.133
Bukombe	Tanzania	0.748	0.468	1.194	-0.055	0.643
Chato	Tanzania	0.741	0.464	1.184	-0.082	0.731
Geita	Tanzania	1.323	0.829	2.112	0.261	0.589
Mbogwe	Tanzania	0.808	0.506	1.290	0.044	0.385
Nyang'wale	Tanzania	0.804	0.504	1.283	0.138	0.163
Iringa	Tanzania	-0.473	-0.297	-0.756	0.066	-0.971
Iringa Urban	Tanzania	0.001	0.000	0.001	0.243	-0.975
Kilolo	Tanzania	-0.276	-0.173	-0.441	-0.095	-0.709
Mufindi	Tanzania	-0.524	-0.328	-0.837	0.070	-1.007
Biharamulo	Tanzania	0.890	0.557	1.421	0.000	0.944
Bukoba	Tanzania	0.563	0.352	0.898	-0.035	0.778

Bukoba Urban	Tanzania	0.466	0.292	0.744	0.115	0.150
Kyerwa	Tanzania	0.821	0.514	1.311	0.104	0.912
Missenyi	Tanzania	0.472	0.295	0.753	-0.062	0.856
Muleba	Tanzania	0.407	0.255	0.649	-0.136	0.760
Ngara	Tanzania	1.228	0.769	1.960	0.020	1.304
Mlele	Tanzania	0.829	0.519	1.323	0.203	0.253
Mpanda	Tanzania	0.154	0.096	0.245	-0.160	0.155
Mpanda Urban	Tanzania	-0.094	-0.059	-0.150	-0.054	-0.047
Buhigwe	Tanzania	1.194	0.748	1.906	0.070	0.455
Kakonko	Tanzania	0.602	0.377	0.962	-0.106	0.775
Kasulu	Tanzania	0.874	0.548	1.396	0.185	0.107
Kasulu Township Auth	Tanzania	0.783	0.490	1.250	0.252	-0.302
Kibondo	Tanzania	0.918	0.575	1.466	-0.101	0.777
Kigoma Urban	Tanzania	0.234	0.146	0.373	0.020	-0.253
Kigoma	Tanzania	1.045	0.655	1.668	0.167	0.258
Uvinza	Tanzania	0.904	0.566	1.443	0.242	0.207
Hai	Tanzania	-0.403	-0.253	-0.644	0.213	-1.283
Moshi	Tanzania	-0.633	-0.397	-1.011	0.118	-1.173
Moshi Urban	Tanzania	-0.162	-0.101	-0.258	0.276	-1.370
Mwanga	Tanzania	-0.691	-0.433	-1.102	0.028	-1.000
Rombo	Tanzania	-0.720	-0.451	-1.150	0.016	-1.049
Same	Tanzania	-0.535	-0.335	-0.855	0.209	-1.049
Siha	Tanzania	-0.425	-0.266	-0.679	0.163	-1.256
Kilwa	Tanzania	-0.215	-0.135	-0.344	0.166	-0.889
Lindi	Tanzania	-0.032	-0.020	-0.050	0.030	-0.621
Lindi Urban	Tanzania	-0.220	-0.138	-0.351	0.119	-0.903
Liwale	Tanzania	-0.068	-0.043	-0.108	0.024	-0.559
Nachingwea	Tanzania	-0.335	-0.210	-0.534	0.059	-0.773
Ruangwa	Tanzania	-0.240	-0.151	-0.384	0.053	-0.713
Babati	Tanzania	-0.910	-0.570	-1.453	-0.098	-1.142
Babati Urban	Tanzania	-0.280	-0.176	-0.447	0.216	-1.268
Hanang	Tanzania	-1.007	-0.631	-1.608	-0.068	-1.026
Kiteto	Tanzania	-0.214	-0.134	-0.341	0.496	-1.255
Mbulu	Tanzania	-0.923	-0.578	-1.473	-0.011	-1.025
Bunda TC	Tanzania	-0.055	-0.034	-0.088	-0.294	0.474

Butiama	Tanzania	1.071	0.671	1.710	0.184	0.873
Musoma	Tanzania	0.105	0.066	0.167	-0.024	0.263
Musoma Urban	Tanzania	0.556	0.348	0.887	0.162	0.001
Rorya	Tanzania	0.596	0.373	0.951	-0.059	0.803
Tarime	Tanzania	0.629	0.394	1.004	0.216	0.495
Chunya	Tanzania	-0.205	-0.128	-0.327	0.223	-0.941
Kyela	Tanzania	-0.352	-0.220	-0.562	0.126	-0.839
Mbarali	Tanzania	-0.432	-0.271	-0.690	0.111	-1.059
Mbeya	Tanzania	-0.085	-0.053	-0.136	0.288	-0.918
Mbeya Urban	Tanzania	-0.403	-0.253	-0.644	0.072	-1.004
Mbozi	Tanzania	-0.338	-0.212	-0.540	0.177	-1.114
Momba	Tanzania	-0.428	-0.268	-0.683	0.147	-1.139
Rungwe	Tanzania	-0.409	-0.256	-0.653	0.004	-0.847
Gairo	Tanzania	-0.312	-0.195	-0.498	0.188	-1.041
Kilombero	Tanzania	0.183	0.114	0.291	0.343	-0.707
Kilosa	Tanzania	0.157	0.098	0.250	0.048	-0.452
Morogoro	Tanzania	0.374	0.234	0.597	0.209	-0.530
Morogoro Urban	Tanzania	0.002	0.001	0.004	0.149	-0.759
Mvomero	Tanzania	0.064	0.040	0.102	-0.042	-0.438
Ulanga	Tanzania	0.979	0.613	1.564	0.351	-0.101
Masasi	Tanzania	-0.404	-0.253	-0.644	-0.060	-0.615
Masasi Township Auth	Tanzania	-0.019	-0.012	-0.030	-0.054	-0.124
Mtwara DC	Tanzania	-0.018	-0.011	-0.029	0.316	-1.384
Mtwara Urban	Tanzania	0.153	0.096	0.245	0.245	-1.371
Nanyumbu	Tanzania	-0.419	-0.262	-0.668	0.034	-0.732
Newala DC	Tanzania	-0.479	-0.300	-0.765	-0.069	-0.628
Tandahimba	Tanzania	0.235	0.147	0.375	-0.042	-0.166
Ilemela	Tanzania	0.435	0.272	0.694	0.171	-0.340
Kwimba	Tanzania	0.658	0.412	1.051	0.252	-0.178
Magu	Tanzania	0.730	0.457	1.166	0.416	-0.110
Misungwi	Tanzania	0.687	0.430	1.097	0.114	0.039
Nyamagana	Tanzania	0.737	0.461	1.176	-0.067	0.382
Sengerema DC	Tanzania	0.140	0.088	0.224	-0.067	-0.085
Ludewa	Tanzania	-0.637	-0.399	-1.017	-0.131	-0.469

Makambako Township Auth	Tanzania	-0.664	-0.416	-1.061	0.049	-1.080
Makete	Tanzania	-0.169	-0.106	-0.270	0.254	-0.851
Njombe	Tanzania	-0.748	-0.468	-1.194	-0.073	-0.985
Njombe Urban	Tanzania	-0.754	-0.472	-1.204	-0.073	-0.865
Wanging'ombe	Tanzania	-0.493	-0.309	-0.787	0.183	-1.069
Chalinze	Tanzania	-0.550	-0.344	-0.877	0.179	-1.285
Kibaha	Tanzania	0.133	0.083	0.212	0.133	-0.811
Kibaha Urban	Tanzania	-0.131	-0.082	-0.208	0.071	-1.111
Kisarawe	Tanzania	0.327	0.205	0.522	0.394	-0.997
Mkuranga	Tanzania	0.050	0.032	0.081	0.016	-0.924
Rufiji	Tanzania	-0.124	-0.078	-0.198	-0.176	-0.548
Kalambo	Tanzania	-0.247	-0.155	-0.394	0.224	-1.067
Nkasi	Tanzania	0.461	0.288	0.735	0.032	0.026
Sumbawanga	Tanzania	-0.582	-0.364	-0.929	0.065	-1.087
Sumbawanga Urban	Tanzania	-0.071	-0.044	-0.113	0.243	-0.960
Mbinga	Tanzania	0.129	0.081	0.206	-0.289	0.656
Namtumbo	Tanzania	0.637	0.399	1.017	0.010	0.255
Nyasa	Tanzania	-0.603	-0.378	-0.962	-0.243	-0.050
Tunduru	Tanzania	0.795	0.498	1.270	0.239	0.054
Msalala	Tanzania	0.841	0.527	1.342	0.316	-0.056
Kahama TC	Tanzania	0.831	0.520	1.326	0.257	-0.019
Kishapu	Tanzania	0.058	0.036	0.092	0.245	-0.623
Shinyanga	Tanzania	0.226	0.141	0.360	0.196	-0.504
Shinyanga Urban	Tanzania	0.181	0.113	0.288	0.210	-0.807
Bariadi DC	Tanzania	0.651	0.408	1.040	-0.031	0.631
Busega	Tanzania	0.147	0.092	0.234	0.023	0.036
Itilima	Tanzania	0.371	0.232	0.592	0.304	-0.176
Maswa	Tanzania	0.338	0.212	0.539	-0.059	0.054
Meatu	Tanzania	-0.214	-0.134	-0.341	-0.353	-0.002
Ikungi	Tanzania	-0.949	-0.595	-1.515	-0.271	-0.660
Iramba	Tanzania	-0.707	-0.443	-1.129	-0.052	-0.732
Mkalama	Tanzania	-0.874	-0.547	-1.395	-0.078	-0.790
Singida	Tanzania	-0.872	-0.546	-1.392	0.108	-0.956
Singida Urban	Tanzania	-1.198	-0.750	-1.913	-0.078	-0.868

Igunga	Tanzania	-0.224	-0.140	-0.357	0.237	-0.793
Kaliua	Tanzania	1.002	0.628	1.600	0.196	0.463
Sikonge	Tanzania	0.103	0.064	0.164	-0.196	0.075
Tabora Urban	Tanzania	0.053	0.033	0.085	0.061	-0.298
Urambo	Tanzania	0.421	0.263	0.671	-0.008	0.205
Uyui	Tanzania	0.187	0.117	0.299	-0.052	-0.009
Handeni	Tanzania	-0.668	-0.419	-1.067	0.032	-1.125
Handeni Mjini	Tanzania	-0.809	-0.507	-1.291	-0.052	-0.883
Kilindi	Tanzania	-0.163	-0.102	-0.261	-0.135	-0.515
Korogwe	Tanzania	-0.542	-0.340	-0.866	0.189	-1.150
Korogwe Township Auth	Tanzania	-1.039	-0.651	-1.659	0.038	-1.439
Lushoto	Tanzania	-0.873	-0.547	-1.394	-0.006	-1.008
Mkinga	Tanzania	-1.096	-0.687	-1.750	-0.041	-1.247
Muheza	Tanzania	-0.810	-0.507	-1.293	0.188	-1.474
Pangani	Tanzania	-0.970	-0.607	-1.548	0.016	-1.434
Tanga Urban	Tanzania	-0.874	-0.548	-1.395	0.218	-1.702
Arusha	Tanzania	-0.324	-0.203	-0.517	0.242	-1.314
Nsimbo	Tanzania	0.207	0.130	0.331	-0.058	0.067
Busokelo	Tanzania	0.128	0.080	0.205	0.085	-0.473
Bumbuli	Tanzania	-0.881	-0.552	-1.406	0.087	-1.325
Serengeti	Tanzania	0.312	0.195	0.498	-0.230	0.707
Simanjiro	Tanzania	-0.332	-0.208	-0.530	0.481	-1.240
Tarime TC	Tanzania	0.162	0.101	0.258	0.072	0.034
Bunda DC	Tanzania	0.009	0.006	0.014	-0.268	0.552
Bariadi TC	Tanzania	-0.457	-0.286	-0.729	-0.288	0.130
Geita Town	Tanzania	0.002	0.001	0.003	-0.154	-0.008
Manyoni	Tanzania	0.236	0.148	0.377	-0.204	0.287
Ubungo MC	Tanzania	-0.149	-0.093	-0.237	0.259	-1.529
Temeke	Tanzania	0.407	0.255	0.649	0.356	-1.149
Newala TC	Tanzania	0.324	0.203	0.517	0.160	-0.218
Nanyamba TC	Tanzania	-0.237	-0.148	-0.378	0.301	-1.346
Ifakara	Tanzania	-0.099	-0.062	-0.159	0.066	-0.739
Mafinga Township Auth	Tanzania	-0.692	-0.434	-1.105	0.035	-1.019
Mbinga TC	Tanzania	0.400	0.251	0.639	0.210	0.212
Mbulu TC	Tanzania	-0.589	-0.369	-0.941	0.160	-1.049

Songwe	Tanzania	-0.220	-0.138	-0.351	0.083	-0.750
Mpimbwe	Tanzania	-0.740	-0.464	-1.182	-0.049	-0.948
Karagwe	Tanzania	-0.017	-0.011	-0.027	-0.284	0.602
Ileje	Tanzania	-0.180	-0.113	-0.288	0.130	-0.822
Kibiti	Tanzania	-0.140	-0.088	-0.223	0.195	-1.152
Ushetu	Tanzania	1.037	0.650	1.655	0.207	0.404
Buchosa DC	Tanzania	0.624	0.391	0.997	0.148	-0.027
Kondoa	Tanzania	-0.668	-0.418	-1.066	0.139	-1.195
Nzega	Tanzania	0.201	0.126	0.320	-0.112	-0.078
Abim	Uganda	0.773	0.484	1.234	-0.234	1.467
Adjumani	Uganda	-0.598	-0.374	-0.954	-0.295	-0.171
Agago	Uganda	0.881	0.552	1.407	0.197	0.933
Alebtong	Uganda	1.067	0.668	1.703	-0.009	1.288
Amolatar	Uganda	0.665	0.416	1.061	-0.149	0.885
Amudat	Uganda	0.288	0.181	0.460	-0.275	1.259
Amuria	Uganda	1.204	0.754	1.922	0.137	1.342
Amuru	Uganda	0.950	0.595	1.517	0.367	0.417
Apac	Uganda	1.410	0.883	2.251	0.362	0.959
Arua	Uganda	0.181	0.113	0.289	0.103	-0.163
Budaka	Uganda	0.330	0.207	0.527	-0.165	1.155
Bududa	Uganda	-0.287	-0.180	-0.459	-0.202	0.678
Bugiri	Uganda	0.348	0.218	0.555	-0.171	0.937
Buhweju	Uganda	-0.579	-0.363	-0.924	-0.048	0.648
Buikwe	Uganda	0.828	0.519	1.322	0.115	0.668
Bukedea	Uganda	0.376	0.236	0.601	-0.277	1.333
Bukomansimbi	Uganda	0.701	0.439	1.119	0.102	0.729
Bukwo	Uganda	0.256	0.160	0.409	-0.036	0.902
Bulambuli	Uganda	-0.038	-0.024	-0.061	-0.094	0.755
Buliisa	Uganda	0.660	0.414	1.054	0.024	0.576
Bundibugyo	Uganda	0.139	0.087	0.221	0.244	0.286
Bushenyi	Uganda	-0.731	-0.458	-1.167	-0.470	1.078
BusiaU	Uganda	1.083	0.678	1.729	0.011	1.432
Butaleja	Uganda	-0.326	-0.204	-0.521	-0.221	0.537
Butambala	Uganda	-0.026	-0.016	-0.042	0.023	-0.102
Buvuma	Uganda	0.970	0.608	1.549	-0.046	1.095

Buyende	Uganda	1.185	0.742	1.892	0.036	1.223
Dokolo	Uganda	-0.292	-0.183	-0.467	-0.269	0.305
Gomba	Uganda	0.332	0.208	0.530	0.087	0.286
Gulu	Uganda	0.939	0.588	1.499	0.033	0.882
Hoima	Uganda	-0.273	-0.171	-0.436	-0.337	0.384
Ibanda	Uganda	0.036	0.023	0.057	0.040	0.863
Iganga	Uganda	0.854	0.535	1.363	0.099	0.937
Isingiro	Uganda	0.048	0.030	0.077	0.170	0.473
Jinja	Uganda	0.943	0.591	1.505	0.094	0.784
Kaabong	Uganda	0.838	0.525	1.337	-0.144	1.634
Kabale	Uganda	-0.933	-0.584	-1.489	-0.087	0.072
Kabarole	Uganda	-0.434	-0.272	-0.693	-0.169	0.268
Kaberamaido	Uganda	0.238	0.149	0.381	0.115	0.426
Kagadi	Uganda	0.239	0.150	0.381	0.145	0.326
Kakumiro	Uganda	0.149	0.093	0.238	-0.025	0.384
Kalangala	Uganda	-0.125	-0.078	-0.199	-0.107	0.108
Kaliro	Uganda	1.325	0.830	2.115	0.279	1.195
Kalungu	Uganda	0.482	0.302	0.769	0.036	0.414
Kampala	Uganda	0.495	0.310	0.790	0.163	0.015
Kamuli	Uganda	1.236	0.774	1.973	0.058	1.083
Kamwenge	Uganda	-0.266	-0.167	-0.424	-0.087	0.512
Kanungu	Uganda	-0.785	-0.492	-1.254	-0.223	0.555
Kapchorwa	Uganda	-0.280	-0.175	-0.446	-0.067	0.402
Kasese	Uganda	-0.324	-0.203	-0.517	-0.412	0.895
Katakwi	Uganda	1.374	0.861	2.193	0.083	1.667
Kayunga	Uganda	1.103	0.691	1.761	0.432	0.582
Kibaale	Uganda	0.244	0.153	0.389	0.053	0.346
Kiboga	Uganda	-0.195	-0.122	-0.311	-0.089	0.028
Kibuku	Uganda	-0.265	-0.166	-0.422	-0.220	0.587
Kiruhura	Uganda	-0.800	-0.501	-1.276	-0.690	0.667
Kiryandongo	Uganda	0.592	0.371	0.945	0.020	0.514
Kisoro	Uganda	-1.128	-0.707	-1.801	-0.173	0.159
Kitgum	Uganda	0.985	0.617	1.572	-0.053	1.354
Koboko	Uganda	-0.403	-0.253	-0.644	0.033	-0.894
Kole	Uganda	0.876	0.549	1.399	0.166	0.605

Kotido	Uganda	1.091	0.683	1.742	-0.177	1.774
Kumi	Uganda	0.566	0.354	0.903	-0.200	1.361
Kween	Uganda	-0.225	-0.141	-0.358	-0.206	0.704
Kyankwanzi	Uganda	-0.074	-0.047	-0.119	-0.008	0.103
Kyegegwa	Uganda	0.252	0.158	0.402	0.042	0.543
Kyenjojo	Uganda	0.408	0.255	0.651	0.283	0.296
Lamwo	Uganda	0.654	0.410	1.045	-0.143	0.949
Lira	Uganda	0.743	0.465	1.186	-0.281	1.167
Luuka	Uganda	1.411	0.884	2.253	0.055	1.361
Luwero	Uganda	0.295	0.185	0.470	-0.104	0.424
Lwengo	Uganda	0.730	0.457	1.165	-0.245	1.336
Lyantonde	Uganda	0.475	0.297	0.758	-0.151	1.246
Maracha	Uganda	0.263	0.165	0.420	0.261	-0.578
Masaka	Uganda	0.121	0.076	0.194	-0.047	0.356
Masindi	Uganda	0.427	0.267	0.681	0.189	0.236
Mayuge	Uganda	0.911	0.571	1.454	-0.226	1.315
Mbale	Uganda	0.293	0.184	0.468	-0.070	0.983
Mbarara	Uganda	-0.650	-0.407	-1.037	-0.459	0.889
Mitooma	Uganda	-0.462	-0.289	-0.737	-0.291	1.136
Mityana	Uganda	-0.052	-0.032	-0.083	0.103	-0.138
Moroto	Uganda	0.509	0.319	0.812	-0.411	1.594
Moyo	Uganda	-0.197	-0.124	-0.315	-0.037	-0.263
Mpigi	Uganda	-0.010	-0.006	-0.016	0.113	-0.151
Mubende	Uganda	0.175	0.110	0.280	-0.091	0.431
Mukono	Uganda	0.324	0.203	0.518	-0.101	0.399
Nakapiripirit	Uganda	0.887	0.556	1.416	0.067	1.385
Nakaseke	Uganda	-0.115	-0.072	-0.184	-0.070	0.006
Nakasongola	Uganda	0.029	0.018	0.047	-0.151	0.223
Namayingo	Uganda	0.819	0.513	1.308	0.234	0.921
Namutumba	Uganda	-0.279	-0.175	-0.446	-0.237	0.471
Napak	Uganda	1.319	0.826	2.106	-0.050	1.823
Nebbi	Uganda	1.123	0.703	1.793	0.062	0.729
Ngora	Uganda	0.868	0.544	1.385	-0.149	1.519
Ntoroko	Uganda	-0.604	-0.378	-0.963	-0.323	0.264
Ntungamo	Uganda	-0.498	-0.312	-0.796	-0.219	0.778

Nwoya	Uganda	0.698	0.437	1.115	0.151	0.420
Omoro	Uganda	0.969	0.607	1.547	0.040	0.896
Otuke	Uganda	0.323	0.202	0.515	-0.285	1.004
Oyam	Uganda	0.906	0.567	1.446	-0.110	1.012
Pader	Uganda	0.323	0.202	0.515	-0.048	0.644
Pallisa	Uganda	0.075	0.047	0.120	-0.306	0.930
Kyotera	Uganda	0.739	0.463	1.179	0.026	0.974
Rubanda	Uganda	-1.110	-0.695	-1.772	-0.322	0.327
Rubirizi	Uganda	-0.542	-0.340	-0.866	-0.442	0.988
Rukungiri	Uganda	-0.245	-0.154	-0.391	0.088	0.760
Serere	Uganda	-0.110	-0.069	-0.176	-0.225	0.610
Sheema	Uganda	-0.933	-0.584	-1.489	-0.364	0.791
Sironko	Uganda	-0.422	-0.265	-0.674	-0.233	0.608
Soroti	Uganda	0.650	0.407	1.038	-0.164	1.199
Ssembabule	Uganda	0.030	0.019	0.048	0.020	0.422
Tororo	Uganda	-0.534	-0.334	-0.852	-0.105	0.203
Wakiso	Uganda	0.457	0.286	0.729	0.152	0.218
Yumbe	Uganda	0.197	0.123	0.314	0.143	-0.368
Zombo	Uganda	0.858	0.538	1.370	0.111	0.406
Pakwach	Uganda	0.717	0.449	1.145	0.089	0.382
Rakai	Uganda	0.497	0.311	0.793	-0.095	1.104
Rukiga	Uganda	-0.798	-0.500	-1.274	-0.126	0.358
Bunyangabu	Uganda	-0.156	-0.098	-0.249	-0.056	0.460
Butebo	Uganda	-0.084	-0.053	-0.134	-0.201	0.856
Manafwa	Uganda	0.315	0.197	0.502	0.072	0.795
